# Congratulatory Message from the President of International Commission on Occupational Health

**DOI:** 10.1186/2052-4374-25-2

**Published:** 2013-05-21

**Authors:** Kazutaka Kogi

**Affiliations:** 1 International Commission on Occupational Health, Kawasaki, Japan

## 

Heartfelt congratulations on the inauguration of a new journal addressing the field of occupational and environmental medicine. It is opportune to provide an interactive forum for research and practice in this field, at the time of globalizing economy and rapid changes in our work and life. The initiative of the Korean Society of Occupational and Environmental Medicine in response to the current broad-ranging developments is highly welcome.

As strategic goals of our joint action, the International Commission on Occupational Health (ICOH) emphasizes international and regional collaboration in promoting proactive risk assessment and control and in extending effective and basic occupational health services for all workers. In increasingly diversifying work situations, it is essential to focus on comprehensive evidence-informed risk management supported by practical action-oriented procedures that have real impact in the local context. The networking of good-practice approaches in participatory work life improvement processes is particularly important. The scientific developments in this regard are remarkable in all regions of the world. The launch of this journal surely gives new impetus for developing a culture of sustainable prevention in the workplace and in the community, reflecting the advances in this collaboration.

We are confident the journal is going to make a significant contribution to the quality of research and practice aimed at our common goals. We are pleased to join our international partners in supporting the journal.

The author, Kazutaka Kogi, has given permission for his photo to be used as the cover page.

